# Long-term Results of Duodeno-jejunal Bypass in the Treatment of Obesity and Type 2 Diabetes

**DOI:** 10.1007/s11695-023-06979-4

**Published:** 2023-12-19

**Authors:** Jan Kral, Marek Benes, Vera Lanska, Peter Macinga, Pavel Drastich, Julius Spicak, Tomas Hucl

**Affiliations:** 1 Department of Hepatogastroenterology, Institute for Clinical and Experimental Medicine, Videnska 1958/9, 140 21, Prague, Czech Republic; 2 Department of Internal Medicine, Second Faculty of Medicine, Charles University, Prague, Czech Republic; 3 Department of Statistics, Institute for Clinical and Experimental Medicine, Videnska 1958/9, 140 21, Prague, Czech Republic

**Keywords:** Bariatric, Obesity, Type 2 diabetes mellitus, Endoscopy, Duodenal-jejunal bypass

## Abstract

**Purpose:**

Obesity and its related severe comorbidities are increasing rapidly. The duodenal-jejunal bypass is an endoscopically implanted device (mimicking the Roux-en-Y gastric bypass) developed to support weight reduction and improve type 2 diabetes control.

**Materials and Methods:**

Retrospective data analysis of consecutive patients undergoing duodenal-jejunal bypass (EndoBarrier®, DJB) implantation between 2013 and 2017 was performed to evaluate safety as well as short- and long-term efficacy.

**Results:**

One hundred and twenty-one patients (mean BMI of 43.1 ± 7.2 kg/m^2^ and weight of 138.2 ± 28.6 kg) underwent DJB implantation. The mean dwelling time was 15.5 months, the mean total body weight loss (%TBWL) after explantation was 10.3% ± 7.9% (14.2 kg, *p* < 0.0001), and the mean BMI was 39.5 ± 7.3 kg/m^2^ (*p* < 0.0001). There was no significant weight gain 24 months after the explantation. Seventy-seven patients had type 2 diabetes mellitus (T2DM) with a mean HbA1c before implantation of 5.6% (*n* = 52). The mean HbA1c after explantation was 5.1% (*p* = 0.0001). Significant reductions in transaminase and lipid levels before and after explantation were observed. One complication occurred during implantation and another during explantation. In 16 patients, the device had to be extracted earlier than expected (7 for severe adverse events and 9 for adverse events; 13.2%).

**Conclusion:**

Despite an evident rate of adverse events, the DJB shows promise as a weight-loss procedure. Our results show that some patients implanted with the device maintained reduced weight even 24 months after explantation, while many improved T2DM control.

**Graphical Abstract:**

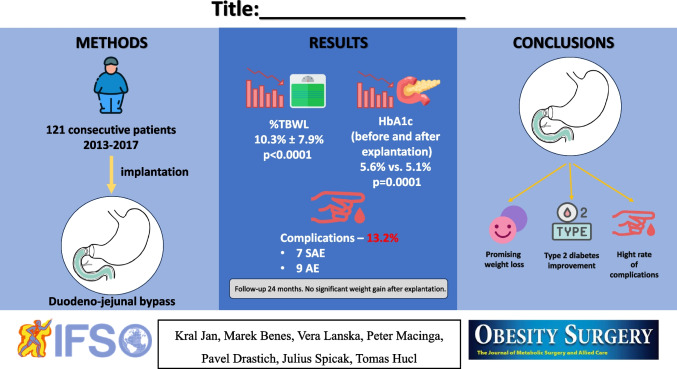

**Supplementary Information:**

The online version contains supplementary material available at 10.1007/s11695-023-06979-4.

## Introduction

Overweight and obesity are challenging conditions that affect millions of people worldwide. Despite the considerable measures taken to fight obesity, the number of patients with the condition continues to increase rapidly. In 2019, the estimated percentage of overweight and obese individuals in the European population was 53% and 17%, respectively [1]. The prevalence of obesity in the United States (US) between 2017 and 2020 was 41.9% [2]. Despite concerted efforts to curb obesity, its prevalence continues to surge. The financial toll of obesity and its associated complications is staggering, estimated to be approximately 147 billion US dollars annually in the US and around 70 billion Euros per year in Europe [2–4]. The economic repercussions of obesity are significant and widespread, unaffected by a country's economic status or geographical location. If current trends persist, these impacts will likely escalate over time [5].

A sedentary lifestyle, processed food, a high-calorie diet, low physical activity, economic growth, and industrialization are the major factors that contribute to the growing increase in incidence and consequent impaired quality of life. Obesity and overweight are closely connected to severe comorbidities such as type 2 diabetes (T2DM), hypertension, myocardial infarction, stroke, fatty liver disease, depression, and cancer [6–8].

Bariatric procedures such as intragastric balloons, laparoscopic sleeve gastrectomy, and the Roux-en-Y gastric bypass (RYGB) are effective methods for treating obese patients and reducing the impact of obesity-related comorbidities [9–11]. Despite of the effect of bariatric procedures, new effective alternatives to these procedures are still needed. EndoBarrier® (EB) is an endoscopically placed device (consisting of a flexible 60-cm sleeve) that mimics the RYGB bypass. The underlying hypothesis of the EB technology is to emulate the effects of bariatric bypass surgery without the necessity of undergoing an actual surgical operation. It is postulated that this device can lead to a reduction in nutrient absorption, changes in gut hormones, and shifts in the composition of the gut mikrobiota. Based on the findings of previous studies, EB (duodeno-jejunal bypass) appears an effective procedure for supporting weight loss and improving glycaemic control DM patients. Studies have also shown a non-negligible risk of possible complications [12–14]. Our aim was to evaluate the effectiveness and safety of the duodenal-jejunal bypass (DJB) in the treatment of obesity and T2DM.

## Methods

### Design and Population

Patient data were collected retrospectively. We analyzed 121 patients consecutively implanted with a DJB device (EB, GI Dynamics, Boston, USA) between 2013 and 2017. Our study was primarily aimed at assessing the safety and efficacy of the device. Main inclusion criteria was BMI ≥ 27 kg/m^2^. All participants gave their informed signed consent. The study was authorized by the ethics committee.

### Data Management and Statistical Analysis

Data on patient demographics, anthropometric factors, age, gender, body weight, body mass index (BMI), waist circumference, and %TBWL before implantation and at the time of explantation were collected. Endoscopic procedure protocols as well as complications during placement and extraction of the implanted device were monitored. Patients were followed for 2 years.

Statistical analysis was performed using JMP®, version 16 (SAS Institute Inc., Cary, NC, USA). Descriptive statistics consisted of absolute and relative frequencies as well as mean with standard deviation. Differences between time periods were compared using McNemar’s test (categorical parameters) and the one-sample Wilcoxon or paired t-test (continuous parameters). Relations between continuous variables were measured using Spearman’s rank correlation coefficient. Results with a two-sided p-value < 0.05 were considered statistically significant.

### Device Effectiveness and Safety

EB effectiveness was determined according to %TBWL and changes in BMI. For patients with T2DM, changes in HbA1c were used. We documented all adverse events occurring during device implantation and explantation. Adverse events were subsequently graded according to the Common Terminology Criteria for Adverse Events (CTCAE), version 5.0 [15].

### Device Implantation/Explantation Procedure

**Fig. 1:**
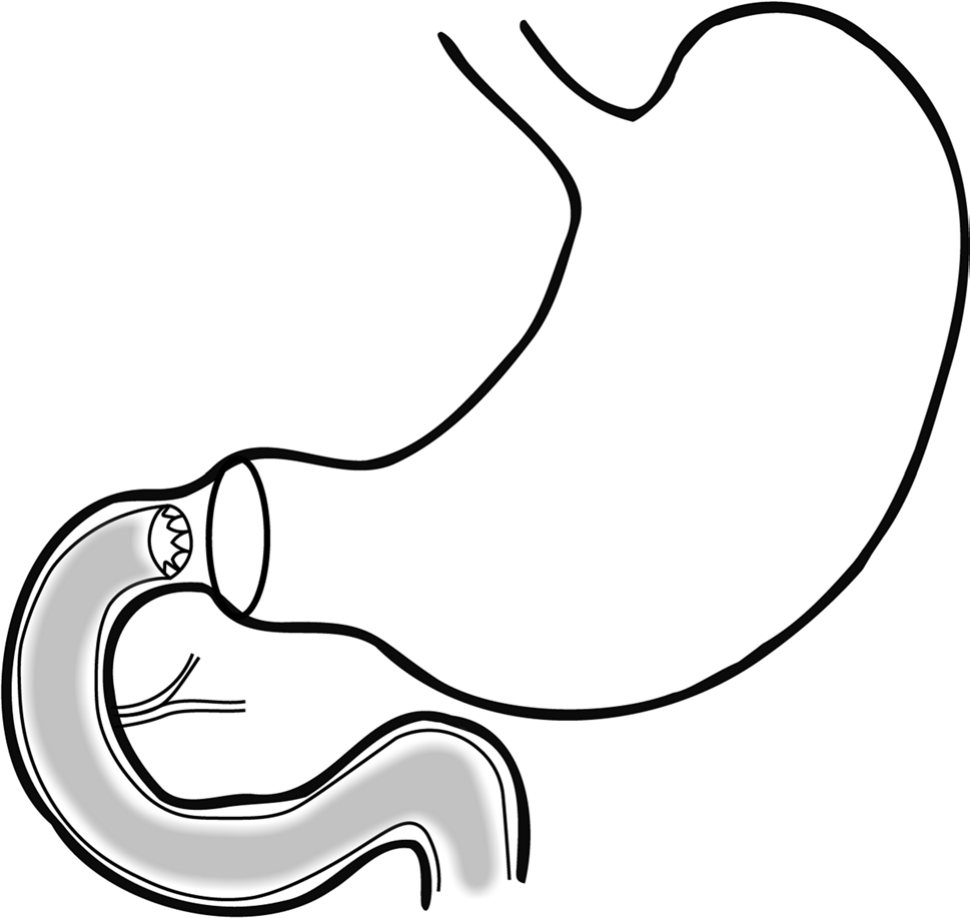
EndoBarrier placement diagram

The DJB was implanted by three specialist endoscopists (ten years of experience each). Before implantation, an upper GI endoscopy was performed to ensure no contraindication for implantation. A 60 cm long thin plastic liner is loaded preloaded in a deliver system, which is introduced over a guidewire into the duodenum bulb according to protocol instructions. The device is expanded down to the proximal jejunum. The device is fixed in the duodenum bulb via a metal ring at the its upper end (Fig. 1.). General anesthesia was administered with propofol, under the supervision of an anesthesiologist and an anesthesia nurse. The average duration of the procedure was approximately 50 min. The position of the device was confirmed by endoscopy and fluoroscopy. The device was initially implanted for an expected duration of 12 months. Upon completion of this period, it was removed under general anesthesia, a procedure that typically lasted an average of 25 min. All patients received education on nutrition and were instructed to follow a suitable diet following device implantation. Proton pump inhibitors (PPIs) were started prior to implantation and continued for the entire duration of the DJB implantation until one week after explantation. This preventive measure aimed to reduce the risk ofcomplications from mucosal injury.

## Results

We retrospectively analyzed all consecutive patients implanted with an DJB between 2013 and 2017. According to standard protocol, a dwelling time of 12 months was prescribed. Our group comprised a total of 121 patients (89 men and 32 women), with a mean age of 51 years. The mean follow-up were 24 months. Complete demographic data are given in Table 1.

**Table 1:** Basic demographics of the study population

Basic demographics – baseline	Number of patients (*n* = 121)	%
Men	89	73.5
Women	32	26.5
Age (mean, years)	51	
Weight before implantation (mean, kg)	138.2	
BMI before implantation (mean, kg/m^2^)	43.1	
Mean dwelling time (in months)	15.5	
Type 2 diabetes mellitus	77	63.6
Complications of type 2 diabetes mellitus	22	28.5
Arterial hypertension	84	69.4
Dyslipidaemia	38	31.4
Thyropathy	17	14.0
Living in urban area	91	75.3
Living in rural area	30	24.7
Alcohol consumption	86	71.0
Active smoking	25	20.6
Education – primary	5	4.2
Education – secondary	58	47.9
Education – university	58	47.9

The mean DJB dwelling time was 15.5 months (min = 0.2, max = 66.6). One hundred patients completed or exceeded the 12-month dwelling period (82.6%). Of these, 14 patients reached 24 months (11.6%). One patient decided to continue using the DJB device (0.8%), despite medical advice. In 21 patients (17.3%), the liner was extracted before the 12-month period: 9 due to adverse events (dyspepsia, abdominal discomfort; 7.4%), 7 due to severe adverse events (5.8%), and 5 by request due to low weight loss (4.1%).

## Weight Control

**Graph 1:**
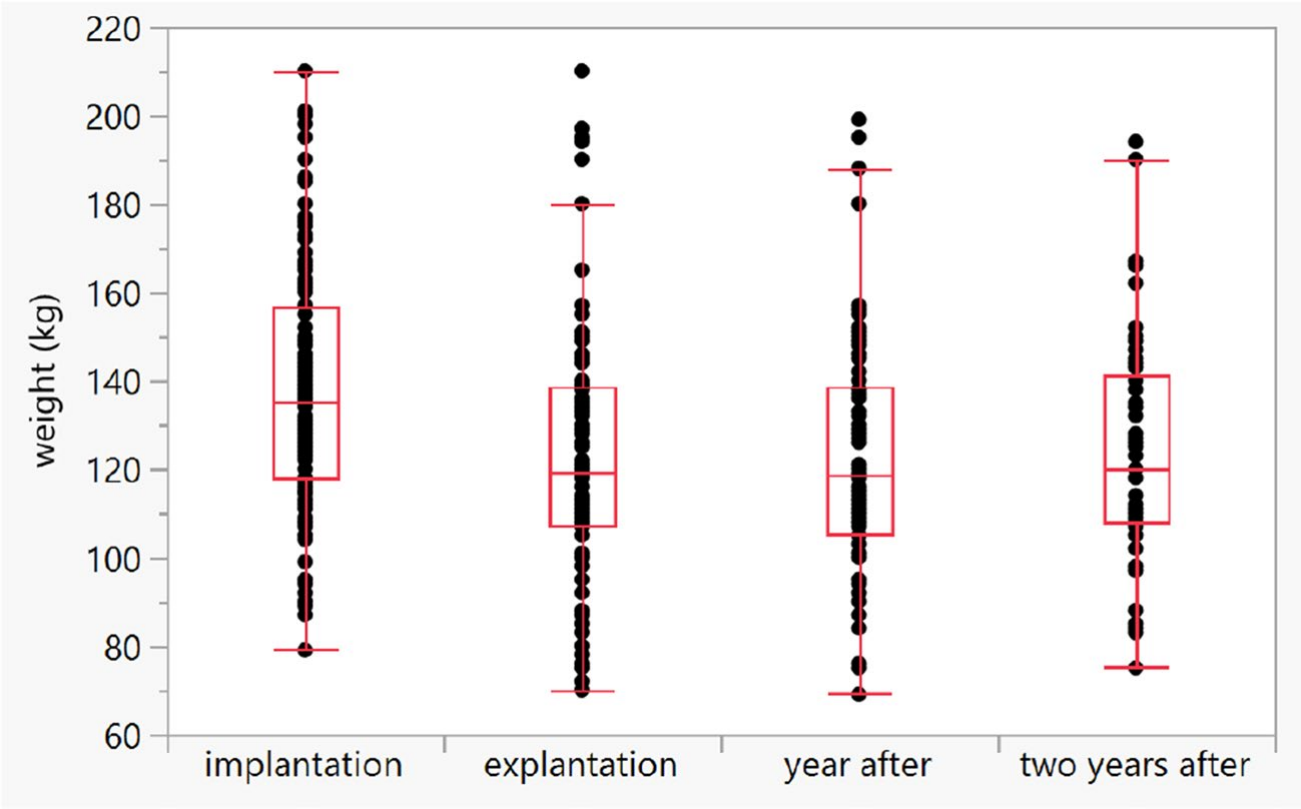
Sustained weight loss after DJB explantation over time (*p* < 0.0001)

The mean weight before DJB implantation was 138.2 ± 28.6 kg (range 79–210) and the mean BMI was 44.1 ± 7.2 kg/m^2^ (range 29.5–62.0). The mean weight at the time of device explantation was 123.9 ± 28.8 kg (range 70–210), the mean BMI was 39.5 ± 7.3 kg/m^2^ (range 26.4–64.8, mean difference: -4.6 ± 0.34 kg/m^2^; *p* < 0.0001), and the mean weight change was 14.2 ± 11.6 kg (*p* < 0.0001). The mean %TBWL was 10.3% ± 7.9% (14.2 kg, *p* < 0.0001). The mean weight one year after implantation was 122.2 ± 27.1 kg. The mean difference between the weight before implantation and the weight one year after implantation was -13.3 ± 11.2 kg (9.6%). The mean weight two years after implantation was 124.2 ± 25.5 kg. The weight loss remained almost the same two years after explantation (-11.1 ± 20.3 kg, *p* < 0.0001; Graph 1) and there was only small weight gain which was not significant two years after explantation (mean weight after 1 year 118.5 kg vs. 120 kg after 2 years; p > 0.05). There was a significant correlation between dwelling time (in days) and weight reduction (Spearman ρ = 0.31, *p* < 0.0001). There was no correlation between weight reduction and educational intervention (*p* = 0.35) or patients with/without T2DM (*p* = 0.88).

## Type 2 Diabetes Mellitus

T2DM was present in 77 patients (63.6%); 22 patients (28.5%) had existing complications of T2DM. The mean HbA1c before device implantation was 56.8 ± 20.1 mmol/mol (5.68%) (range 33–114, *n* = 52). Mean glycaemia before implantation was 8.6 ± 3.4 mmol/l (min. 4.75 mmol/l, max. 24.6 mmol/l; *n* = 54). Thirty two T2DM patients finished the 24 month follow-up. Detailed data on T2DM treatment are given in Table 2.

**Table 2:** T2DM treatment

T2DM treatment	*n* = 77	%
Diet	12	15.6
Peroral antidiabetics (PAD)	43	55.8
Insulin	13	16.9
Insulin + PAD	9	11.7

**Graph 2:**
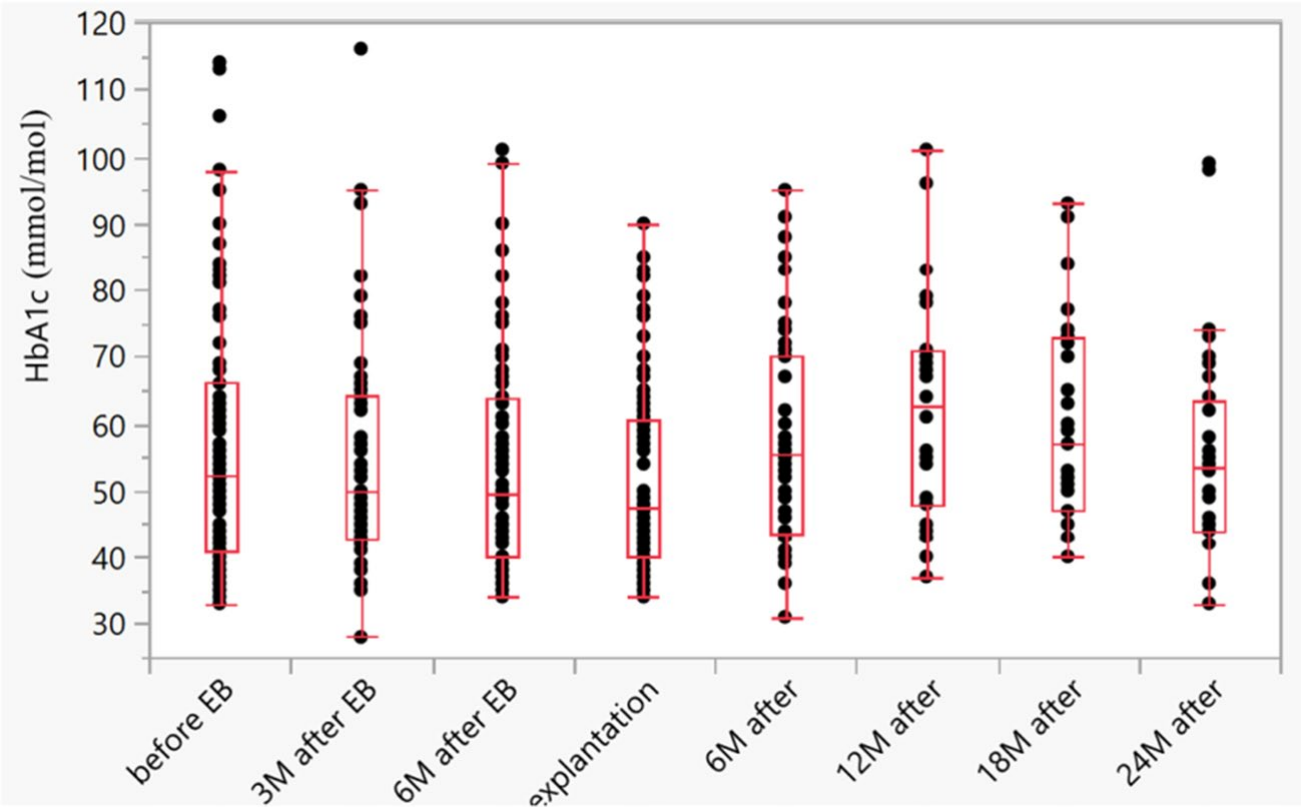
Reduction in HbA1c (mmol/mol) before implantation and after explantation (*p* = 0.0001)

Three months after device implantation, the mean HbA1c level was 5.4% ± 1.6%. Six months after device implantation, the mean HbA1c level was 5.3% ± 1.5%. At device explantation, the mean HbA1c level was 5.1% ± 1.4%. The mean glycaemia level at explantation was 7.5 ± 2.4 mmol/l (min. 4.7 mmol/l, max. 15.3 mmol/l). The mean level of HbA1c 6, 12, 18, and 24 months after explantation was 5.7% ± 1.6%, 6.2% ± 1.5%, 6.0% ± 1.5%, and 5.5% ± 1.5%, respectively (Table 3) (Graph 2). There was a significant reduction in HbA1c before device implantation and after device explantation (*p* = 0.0001; *n* = 52).

**Table 3:** HbA1c and glycaemia levels in patients with T2DM

T2DM: HbA1c and glycaemia			Follow-up			
	Before implantation	At explantation	6 months (*n* = 46)	12 months (*n* = 30)	18 months (*n* = 38)	24 months (*n* = 32)
Glycaemia (mmol/l, mean) (*n* = 54)	8.6	7.5				
HbA1c (%, mean) (*n* = 52)	5.6	5.1	5.7	6.2	6.0	5.5

## Liver Enzymes and Lipid Panels

Levels of transaminases and serum lipids were examined before and after DJB implantation/explantation, (Supplementary File 1, Tables 1 and 2). There was a significant change in liver enzymes (AST, ALT – *p* < 0.0001, GGT – *p* = 0.0002) and lipid panels (LDL, cholesterol – *p* < 0.0001, HDL – *p* = 0.018, TAG – *p* = 0.013) before implantation and after explantation. There was no significant change in ALP or bilirubin.

## Complications

Only one complication occurred during DJB implantation involving a device that failed to expand properly. The case was resolved with the implantation of a replacement device. Similarly, only one complication occurred during DJB explantation where the proximal part of the device ruptured into two parts, both of which were later successfully extracted. The DJB had to be extracted earlier in 16 cases (13.2%), categorized as adverse events (AE; *n* = 9, 7.4%) and severe adverse events (SAE; *n* = 7, 5.8%) (Table 4). Most of the adverse events were mild (Table 5). Four patients required hospitalization due to bleeding, with two experiencing significant hemorrhaging that necessitated blood transfusions. Another patient presented with mild edematous pancreatitis. One case of obstruction was successfully resolved through the extraction of the device, circumventing the need for surgical intervention. Additionally, one patient developed a sizable liver abscess (9.5 cm), which was addressed through ultrasound-guided percutaneous drainage and antibiotic treatment. Five devices were removed by request due to low weight loss (*n* = 5, 4.1%).

**Table 4:** Early extraction of DJB due to severe adverse events

Adverse events	Number of patients (*n* = 9)
Epigastric pain	4
Nausea	3
Abdominal discomfort	1
Vommiting	1

**Table 5:** Early extraction of DJB due to severe adverse events

Early extraction for SAE	Number of patients (*n* = 7)	
Liver abscess	1	drainage
Obstruction	1	Device extraction
Bleeding	4	2 significat bleeding
Pancreatitis	1	Mild pancreatitis

## Discussion

Endoscopic procedures now play an increasingly important role in obesity treatment, which remains a complex challenge. Endoscopic treatment of obesity results in higher weight reduction than pharmacotherapy and, at the same time, fewer complications compared to the standard surgical approach [4]. Intragastric balloons have demonstrated promising outcomes in weight reduction, achieving around a 15% decrease in comparison to placebos. This method, involving a straightforward implantation and removal process, contrasts with the more commonly performed surgical approach—sleeve gastrectomy [16]. Sleeve gastrectomy tends to result in more substantial weight loss, ranging from 33% up to an impressive 90% [17–19]. Pharmacological therapy (GLP-1 agonists) represent a promising and effective new pharmacological approach to obesity treatment. There may be potential for a combined approach utilizing both therapies [20]. Our retrospective analysis revealed a median %TBWL of 10.3% and a mean weight loss of 14.2 kg (*p* < 0.0001). In 2017, Forner et al. conducted a study involving one of the largest cohorts of patients treated with DJB. In total, 114 devices were implanted between the years 2012 and 2015. Patients were treated for a mean duration of 51.1 weeks (12.7 months), with 24% of patients undergoing early explantation before the prescribed 12-month period (due to adverse events.). The mean total body weight change was 12.0 ± 8.6 kg (*p* < 0.001), the mean BMI change was 4.2 ± 3.2 kg/m^2^ (*p* < 0.001), and the mean %TBWL was 10.5 ± 7.3% [12]. In 2018, Patel et al. published the results of multicentric trial with 45 patients (BMI 30–50 kg/m^2^) recruited, 31 patients (69%) completed the prescribed 12-month dwelling time. Ater twelve months the mean weight loss was 15 kg (*p* < 0.05) and BMI had reduced by 4.9 kg/m^2^ (*p* < 0.005). Results for %TBWL and %EWL were not reported [14]. A randomized controlled trial by Ruban et al. compared differences between DJB patients (*n* = 85) and controls (*n* = 85). At 12 months, 24.2% of patients achieved a minimum 15% TBWL compared to the control group (3.7%) (*p* = 0.001), while 57.6% exceeded a 10% TBWL compared to controls (22.2%). There were no differences in weight loss after 24 months in either group (*p* = 0.76) [21]. The mean weight losses reported in the above studies were comparable.

In our study, we observed a reduction in HbA1c and glycaemia. The reduction in HbA1c after explantation was significant (5.6% versus 5.1%, *p* < 0.0001). Based on follow-up results, there was a continuous rise in HbA1c of 5.7% and 6.2% at 6 months and 12 months, respectively. Forner et al. reported a mean baseline HbA1c of 6.7 ± 2% in 38 patients with type T2DM (33.3%). At follow-up (14.7 months), the mean HbA1c was 6.6 ± 1.8%, correlating with a mean change of 0.006 ± 0.9% (*p* = 0.971) [12]. Patel et al. observed a significant reduction in HbA1c after 12 months in their group of 45 patients. The mean HbA1c reduction was 0.8% (*p* < 0.05), occurring as early as 3 months after insertion (0.9%). After explantation, HbA1c levels remained stable [14]. Ruban et al. found no significant reduction in HbA1c in either of their groups at 12 and 24 months (*p* = 0.71) [21]. All of the above studies (except of Forner et al. and Ruban et al.) reported a greater decrease in HbA1c at device explantation. After explantation, a rise in HbA1c was observed across all studies.

In our study, there was a significant reduction in almost all liver enzymes and lipid panels before implantation and after explantation. Forner et al. and Ruban et al. documented significant reduction in liver enzymes and also in lipid metabolism (*p* < 0.001) [12, 21]. In all studies, weight loss was associated with decreased liver enzymes and improved lipid metabolism.

In our patient cohort, 100 patients completed the prescribed 12-month dwelling time, with 14 reaching the 24-month mark. After 2 years of follow-up, the mean weight loss was still significant (-11.1 ± 20.3 kg, *p* < 0.0001). Of the DJB studies published to date, our study boasts the longest follow-up period (despite a fall-off in data) of patients treated with the device. Forner et al. followed patients for a mean of 34 ± 22 weeks after device removal. In patients followed for 6 months after device removal, the mean weight change was 4.5 ± 6.1 kg (*p* = 0.000) [12]. Patel et al. followed patients for six months. After explantation, weight had increased by 2.2 ± 5.1 kg at 3 months (*n* = 31) and by 3.1 ± 5.2 kg at 6 months (*n* = 29) [14]. A study by van Rijn et al. 15 patients were considered eligible for follow-up at a median of 42 months. The %TBWL had increased by only 2.2% at follow-up compared to baseline [22]. Ruban et al. observed no differences in weight loss after 24 months in patients or controls (*p* = 0.76) [21]. Based on the results from our cohort, patients succeeded in maintaining weight loss after device extraction. Even after 24 months, some patients had managed to stabilize their weight.

In our retrospective analysis, one complication occurred during implantation and another during explantation. In 16 cases (13.2%), the DJB had to be extracted earlier due to severe adverse events (*n* = 7, 5.8%) and adverse events (*n* = 9, 7.4%), with another 5 devices extracted by request due to low weight loss (*n* = 5, 4.1%). Of the 114 DJB implantation procedures evaluated by Forner et al., 8 operations were unsuccessful due to 2 cases of active bleeding, 1 respiratory arrest, and 5 cases of incompatible anatomical disposition [12]. In the study by Patel et al., 40 of 45 patients (88.9%) underwent 127 device-related adverse events, most of which were mild (84.4%) [14]. Ruban et al. reported a total of 857 adverse events in 151 patients. Of these, 50 were serious adverse events (migration, upper gastrointestinal bleeding, cholecystitis, liver abscesses, anticoagulation, abdominal pain, withdrawal of consent [21]. The adverse events reported across these studies were usually mild. Nevertheless, the complications associated with the DJB device are far from negligible. The most common complications after gastric bypass reported by Podnos et al. were stomal stenosis (4.73%), bowel obstruction (2.9%), and wound infection (2.98%). Birkmeyer et al. reported overall perioperative complications in 7.3% patients, the most common being after gastric bypass, laparoscopic sleeve gastrectomy and laparoscopic adjustable gastri band [17, 23]. In a review by Mohamed Baraa et al., it was noted that early removal of the intragastric balloon is estimated to occur in approximately 7% of cases. Serious complications such as migration or perforation are less common, occurring in approximately 1.4% and 0.1% of cases, respectively [24].

In 2015, a clinical trial of DJB (the ENDO trial) was stopped by the FDA due to a higher-than-anticipated rate of adverse events (passing the safety threshold of 2% for liver abscesses). The DJB CE mark was subsequently suspended in 2017. The device-related SAE was approximately 9% (varied in clinical trials). However, in general, an SAE rate of more than 1–2% is considered high for endoscopy procedures. Despite these concerns, DJB remains a promising device for the treatment of obesity [25–27]. The enduring effect on weight loss and metabolic function following DJB use still needs to be elucidated. We propose that the time-limited bypass of the small intestine may lead to sustained alterations in gut hormone secretion, creating a significantly different metabolic environment, potentially resulting in the reprogramming of enteroendocrine cells.

## Limitations

The study featured only 77 patients with type 2 diabetes mellitus, while HbA1c and glycaemia were not monitored in all patients during follow-up, making our data less consistent. Another limitation concerns the retrospective nature of the analysis itself, where data were not collected in real-time, the possibility of selection bias, and variation in the duration of the device implant. It's important to bear in mind that 16 DJBs had to be explanted, representing 13.2% of the cases. This fact inevitably imposes certain limitations on our study data. We hypothesize that the modest weight loss observed in our five patients can be ascribed to their nonadherence to the recommended diet and their unmet expectations of swift weight loss. Compliance with dietary advice, close monitoring—an area in which our study was lacking—and the patient's readiness to maintain patience throughout the weight loss journey are key determinants that can profoundly influence the results.

## Conclusions

The DJB, with its mean percentage of %TBWL ranging between 10–15%, could potentially be an effective procedure for weight loss in obesity treatment. Additionally, it may contribute to the metabolic management of type 2 diabetes mellitus, liver enzyme regulation, and lipid metabolism. In our study, we demonstrated that some patients implanted with the device were successful in maintaining weight even after 24 months. In other cases, type 2 DM control, liver enzymes, and lipid profiles all improved. Reflecting safety profile, our study also includes a high percentage of adverse events. In this context, there is still room for improvements in the technical aspects of the device. This could position it between to other procedures such as intragastric balloons and endoscopic sleeve gastroplasty in the continuously evolving field of weight loss treatments, offering not just weight reduction but additional metabolic benefits.

### Supplementary Information

Below is the link to the electronic supplementary material.

Supplementary file1 (DOCX 14 KB)

## Data Availability

Data are available on demand.
