# Supplementary material for: Long-term Results of Duodeno-jejunal Bypass in the Treatment of Obesity and Type 2 Diabetes
Source: Obes Surg. 2023 Dec 19;34(5):1407–14. doi: 10.1007/s11695-023-06979-4 (PMC11031453; doi:10.1007/s11695-023-06979-4)
Supplement: Supplementary file 1 — Supplementary file1 (DOCX 14 KB) [file 11695_2023_6979_MOESM1_ESM.docx]

**Supplementary File 1**

| Liver enzymes (mean) | Before implantation | After explantation |
| --- | --- | --- |
| AST (µkat/l) (ref. 0.17 - 0.75) | 0.56 ± 0.23 | 0.45 ± 0.18 |
| ALT (µkat/l) (ref. 0.17 - 1.17) | 0.88 ± 0.36 | 0.61 ± 0.24 |
| ALP (µkat/l) (ref. 0.58 - 1.75) | 1.36 ± 0.45 | 1.51 ± 0.46 |
| GGT (µkat/l) (ref. 0.15 - 1.07) | 1.12 ± 0.80 | 0.82 ± 0.82 |
| Bilirubin (µmol/l) (ref. 3.4 - 20.0) | 11.3 ± 4.4 | 10.8 ± 5.2 |

**Table 1.** Liver enzymes

| Lipid panel (mean) | Before implantation | After explantation | P value |
| --- | --- | --- | --- |
| TAG (ref. 0.5 - 1.69) | 2.18 ± 1.20 | 2.00 ± 1.76 | **0.013** |
| LDL (ref. 1.2 - 3.0) | 2.93 ± 0.98 | 2.59 ± 0.87 | **<0.0001** |
| HDL (ref. 1.0 - 2.7) | 1.08 ± 0.26 | 1.03 ± 0.25 | **0.019** |
| Total cholesterol (ref. 2.9 - 5.0) | 4.83 ± 1.14 | 4.42 ± 1.01 | **<0.0001** |

**Table 2.** Lipid panel
